# Supplementary material for: Autistic Traits Related to the Importance of Interpersonal Touch and Appreciation of Observed Touch during COVID-19 Social Distancing
Source: Int J Environ Res Public Health. 2023 Sep 11;20(18):6738. doi: 10.3390/ijerph20186738 (PMC10531349; doi:10.3390/ijerph20186738)
Supplement: Supplementary file 1 [file ijerph-20-06738-s001.zip › ijerph-2435135-supplementary.pdf]

Supplementary material I

Interpersonal touch observation

In order to explore the relationship between touch and autistic traits, we assessed whether the pleasantness ratings of touch scores were related to all AQ scores. Using Kendall's tau, we first found that those with lower AQ scores were more likely to appreciate the observed CT-optimal touch as more pleasant, compared to those who scored higher on AQ scores ($N = 377$, $r\tau = -0.283$, $p < 0.001$). Second, we found that those with lower AQ scores were more likely to appreciate the observed CT-non-optimal touch as more pleasant, compared to those who scored higher on AQ scores ($N = 377$, $r\tau = -0.173$, $p < 0.001$). Third, following previous studies, we calculated affective touch awareness as a derivative of the previous scores, by subtracting pleasantness ratings of CT-non-optimal touch from pleasantness ratings of CT-optimal touch. We found that the lower the AQ scores, the higher the affective awareness scores ($N = 377$, $r\tau = -0.192$, $p < 0.001$).

Longing for touch

We explored the relationship between longing for touch and wellbeing during social distancing in relation to autistic traits. First, using Kendall's tau, we found no correlation between AQ scores and the self-report question of being touch frequently enough or not before social distancing ($N = 377$, $r\tau = 0.034$, $p = 0.341$). Second, we explored longing for touch, i.e. preferring to touch others or being touch by others more during social distancing. We found a negative correlation between AQ scores and longing for touch ($N = 377$, $r\tau = -0.288$, $p < 0.001$), where the lower the score on the AQ, the higher the reported amount of longing for touch.
